# Downregulation of RNA binding protein 47 predicts low survival in patients and promotes the development of renal cell malignancies through RNA stability modification

**DOI:** 10.1186/s43556-023-00148-w

**Published:** 2023-11-14

**Authors:** Cheng Wang, Weiquan Li, Xiangui Meng, Hongwei Yuan, Tiexi Yu, Wei Yang, Dong Ni, Lei Liu, Wen Xiao

**Affiliations:** 1 Department of Urology, Tongji Medical College, Union Hospital, Huazhong University of Science and Technology, Wuhan, 430022 China; 2 Institute of Urology, Tongji Medical College, Union Hospital, Huazhong University of Science and Technology, Wuhan, 430022 China

**Keywords:** RNA binding motif protein 47, Clear cell renal carcinoma, Epithelial-mesenchymal transition, RNA stability modification

## Abstract

RNA binding proteins (RBPs) are crucial for cell function, tissue growth, and disease development in disease or normal physiological processes. RNA binding motif protein 47 (RBM47) has been proven to have anti-tumor effects on many cancers, but its effect is not yet clear in renal cancer. Here, we demonstrated the expression and the prognostic role of RBM47 in public databases and clinical samples of clear cell renal carcinoma (ccRCC) with bioinformatics analysis. The possible mechanism of RBM47 in renal cancer was verified by gene function prediction and in vitro experiments. The results showed that RBM47 was downregulated in renal cancers when compared with control groups. Low RBM47 expression indicated poor prognosis in ccRCC. RBM47 expression in renal cancer cell lines was reduced significantly when compared to normal renal tubular epithelial cells. Epithelial-mesenchymal transition (EMT) and transforming growth factor-β signaling pathway was associated with RBM47 in ccRCC by Gene set enrichment analysis. RBM47 expression had a positive correlation with e-cadherin, but a negative correlation with snail and vimentin. RBM47 overexpression could repress the migration, invasion activity, and proliferation capacity of renal cancer cells, while RBM47 inhibition could promote the development of the malignant features through EMT signaling by RNA stability modification. Therefore, our results suggest that RBM47, as a new molecular biomarker, may play a key role in the cancer development of ccRCC.

**Supplementary Information:**

The online version contains supplementary material available at 10.1186/s43556-023-00148-w.

## Introduction

The death rate of renal cell carcinoma (RCC) ranks 16th in the global malignant tumors and the most common subtype is the clear cell type (ccRCC) [1]. It is estimated that there will be approximately 14,890 deaths and 81,800 new cases in America [2], and approximately 46,345 deaths and 77,410 new cases in China in 2022 [3]. Approximately 17% of ccRCC cancer patients experienced distant metastasis at the time of initial diagnosis [4]. The postoperative local recurrence or metastasis rate of patients is 20–40% [5]. The main first-line drug treatment for ccRCC patients is tyrosine kinase inhibitors (TKIs), poor clinical outcomes still appear due to drug resistance of TKIs [6, 7]. Therefore, exploring more effective new prognostic biomarkers and their potential mechanisms is very important [8].

RNA modification, including alternative polyadenylation, N6-methyladenosine (m^6^A), adenosine-to-inosine RNA editing, through multiple RNA modification writers, RNA demethylase can modify RNA stability [9, 10]. RNA binding proteins (RBPs) can control RNA translation efficiency, splicing, stability, and localization by direct binding to RNA, contributing to cancer development [11, 12]. RNA binding motif protein 47 (RBM47) is an RNA binding protein that primarily binds to introns or 3 ‘- UTRs of mRNA [13]. Apobecc1 complementary factor (A1CF) and hnRNP-Q can regulate RNA editing, splicing and transcriptional stability, and they are the closest homologs of RBM47 [14]. Recently, RBM47 has been proven to be a tumor suppressor for colorectal cancer (CRC) [15] and lung cancer [16]. RBM47 can induce targeted RNA partners’ inactivation and inhibit cancer progression through downstream signaling in breast cancer [17]. RBM47 has been discovered as a new p53/p21 signaling regulator in head and neck squamous-cell carcinoma [18]. RBM47 activates autophagy and inhibits papillary thyroid carcinoma (PTC) cell proliferation by directly binding and stabilizing lncRNA small nuclear RNA host gene 5 (SNHG5) [19].

Epithelial mesenchymal metastasis (EMT) plays an important promoting role in the progression and evolution of various cancers [20–25]. Vimentin (VIM), snail, and e-cadherin can contribute to EMT process and promote tumor cell growth [26, 27]. Our previous research showed that stabilizing SET domain bifurcated histone lysine methyltransferase 1 (SETDB1) mRNA could facilitate EMT in bladder cancer [28]. Solute carrier family 27 member 2 (SLC27A2) suppresses renal cancer ability by down-regulating EMT [29]. RBM47 can be directly inhibited during EMT induction, however, the expression of RBM47 and its effect on EMT in ccRCC are still unclear.

Here, we investigated the clinical pathological characteristics and patient survival rate of RBM47 in ccRCC, as well as its function role in renal cancer cells. The expression of RBM47 was downregulated in renal cell carcinoma tissues and cells. High RBM47 expression indicated good progression, and could inhibit EMT of renal cancer cells. Mechanistically, RBM47 might play a tumor suppressive role by regulating the stability of e-cadherin RNA in the EMT signaling pathway in ccRCC.

## Results

### RBM47 is down-regulated significantly in ccRCC tissues

**Fig. 1:**
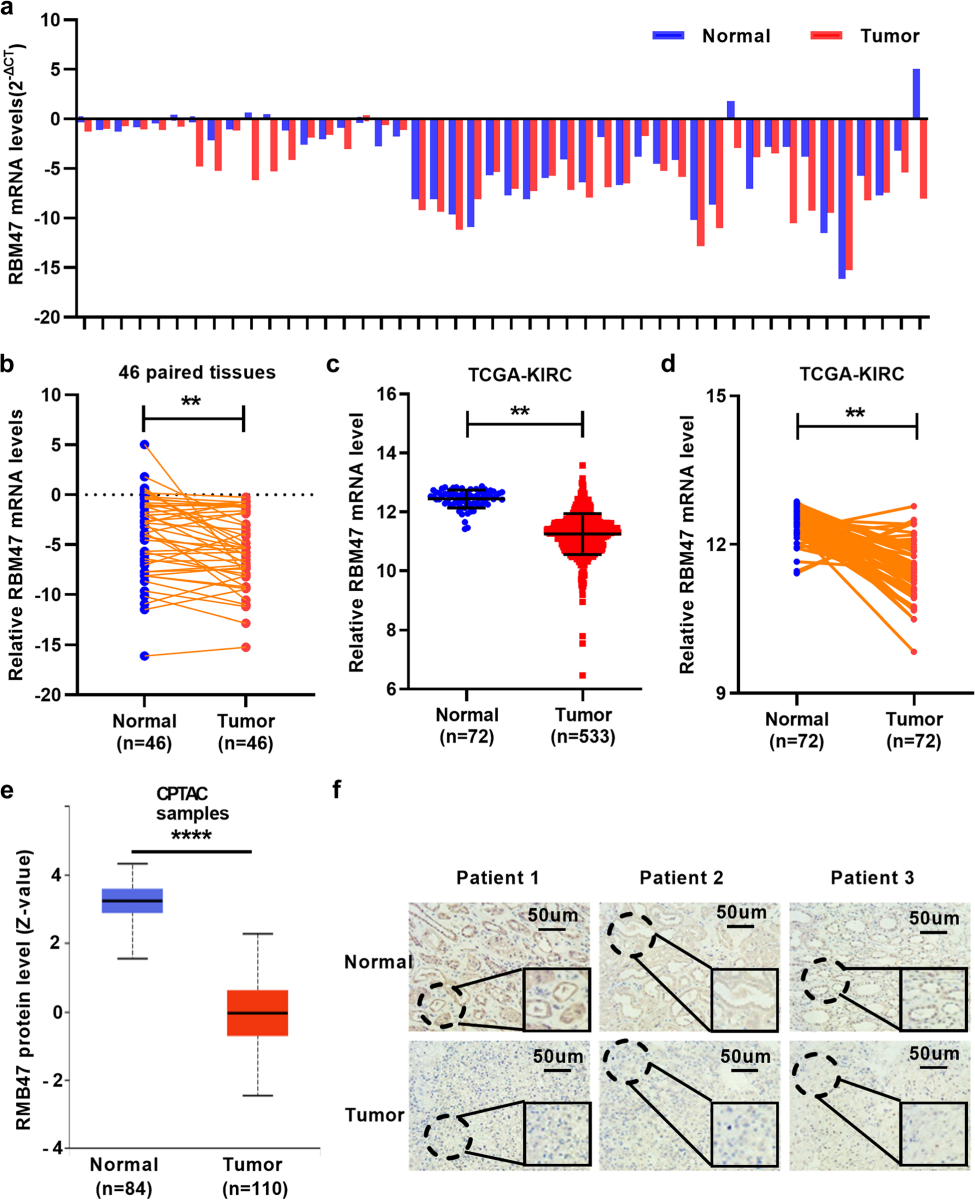
RBM47 expression in ccRCC tissues. **a** and **b** RBM47 was down-regulated in ccRCC cancer tissues. **c** and **d** RBM47 was decreased in ccRCC cancer tissues from TCGA-KIRC dataset. **e** and **f** Protein levels of RBM47 were down-regulated in ccRCC cancer tissues analyzing from public database and clinical samples with immunohistochemistry (IHC). TCGA-KIRC: The Cancer Genome Atlas Clear cell renal carcinoma. Data were showed with means ± standard. **P < 0.01, ****P < 0.0001

Firstly, we explored the differential expression of RBM47 in ccRCC patient tissues with qRT-PCR. The results showed that RBM47 were decreased in ccRCC patients (Fig. 1a and b). Similarly, we found low expression of RBM47 in the Cancer Genome Atlas Kidney Clear Cell Carcinoma (TCGA-KIRC) (Fig. 1c), as well as in 72 paired ccRCC tissues (Fig. 1d), and the correlation between RBM47 mRNA expression and clinicopathological parameters of ccRCC patients was showed in Table 1. Furthermore, the protein levels of RBM47 were decreased in ccRCC cancer tissues in public database (the Clinical Proteomic Tumor Analysis Consortium, CPTAC) and clinical samples with immunohistochemistry (Fig. 1e and f). The results showed a decrease in the expression of RBM47 in renal cancer tissues, indicating that it may have a certain inhibitory effect on renal cell carcinoma.

**Table 1:** Correlation between RBM47 mRNA expression and clinicopathological parameters of ccRCC patients

Variables		RBM47 mRNA expression			
		Low (n = 259)	High (n = 259)	χ^2^	P value
Age (years)	<=60	131	124		
	> 60	128	135	0.378	0.598
Sex	male	179	158		
	female	80	101	3.745	0.065
T stage	T1 + T2	149	180		
	T3 + T4	110	79	8.006	0.006
N stage	N0 + NX	252	250		
	N1	7	9	0.258	0.801
M stage	M0 + MX	213	226		
	M1	46	33	2.524	0.142
Grade	G1 + G2	113	128		
	G3 + G4	146	131	1.746	0.217
TNM stage	I + II	142	170		
	III + IV	117	89	6.319	0.015

### Prognostic role of RBM47 in ccRCC

**Fig. 2:**
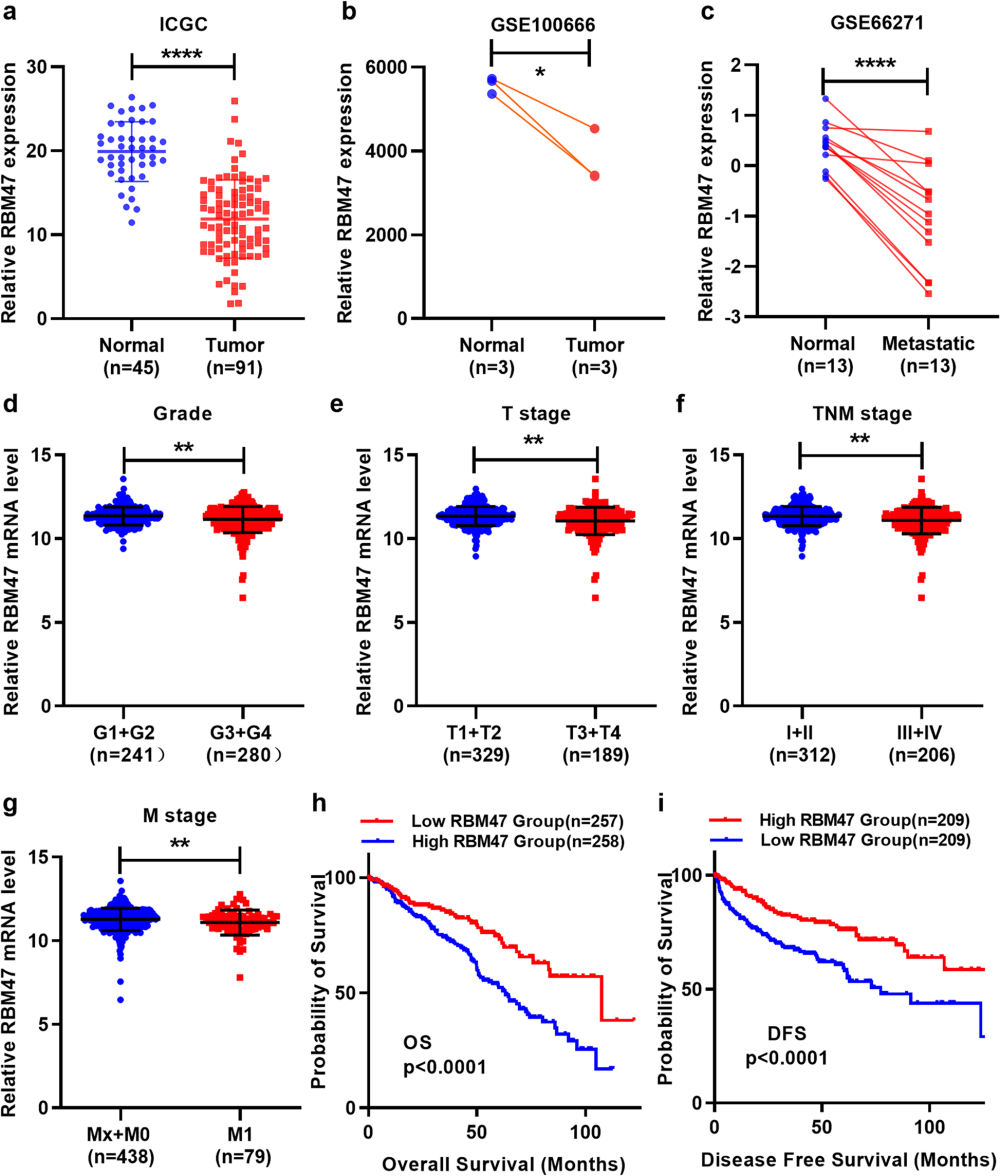
Prognostic effect of RBM47 in ccRCC. **a**-**c** RBM47 was decreased in ccRCC cancer tissues from ICGC and GEO datasets. RBM47 was down-regulated in higher grade (**d**), higher T stage (**e**), higher TNM stage (f), higher M stage (**g**). **h** and **i** High RBM47 expression predicted good overall survival and disease-free survival in ccRCC. GEO: Gene Expression Omnibus; ICGC: International Cancer Genome Consortium. Data were showed with means ± standard. *P < 0.05, **P < 0.01, ****P < 0.0001

Next, we validated the expression of RBM47 in other public databases and found the down-regulation of RBM47 in International Cancer Genome Consortium (ICGC) and Gene Expression Omnibus (GEO) databases (Fig. 2a and c). Then, we investigated the prognostic role of RBM47 in renal cell carcinoma due to the reduced expression of RBM47 in renal cancer. The correlation between clinicopathological parameters and RBM47 expression was explored, and high expression of RBM47 expression had a positive correlation with lower Fuhrman grade, TNM grade, T grade, and M grade (Fig. 2d-g). High RBM47 expression patients had a good overall survival (OS) (Fig. 2h) and disease-free survival (DFS) (Fig. 2i) based on the median RBM47 expression. Furthermore, RBM47 had a prognostic effect in subgroups of ccRCC, including gender, age, T stage, Mx + M0 stage, Nx + N0 stage, Fuhrman G3 + G4 grade, TNM stage, but not N1, M1, or G1 + G2 subgroups (Fig. S1a-l). The same results are also found in DFS of ccRCC: age 60 years, male, T3 + T4 stage, Nx + N0 stage, M stage (Mx + M0, M1), and G3 + G4 grade, but not age ≤ 60 years, female, T1 + T2 stage, N1, M1, or G1 + G2 or TNM stage subgroups (Fig. S2a-i). The results showed that RBM47 can serve as a prognostic marker for renal cancer patients.

### RBM47 inhibits cell proliferation of renal cancer cells

**Fig. 3:**
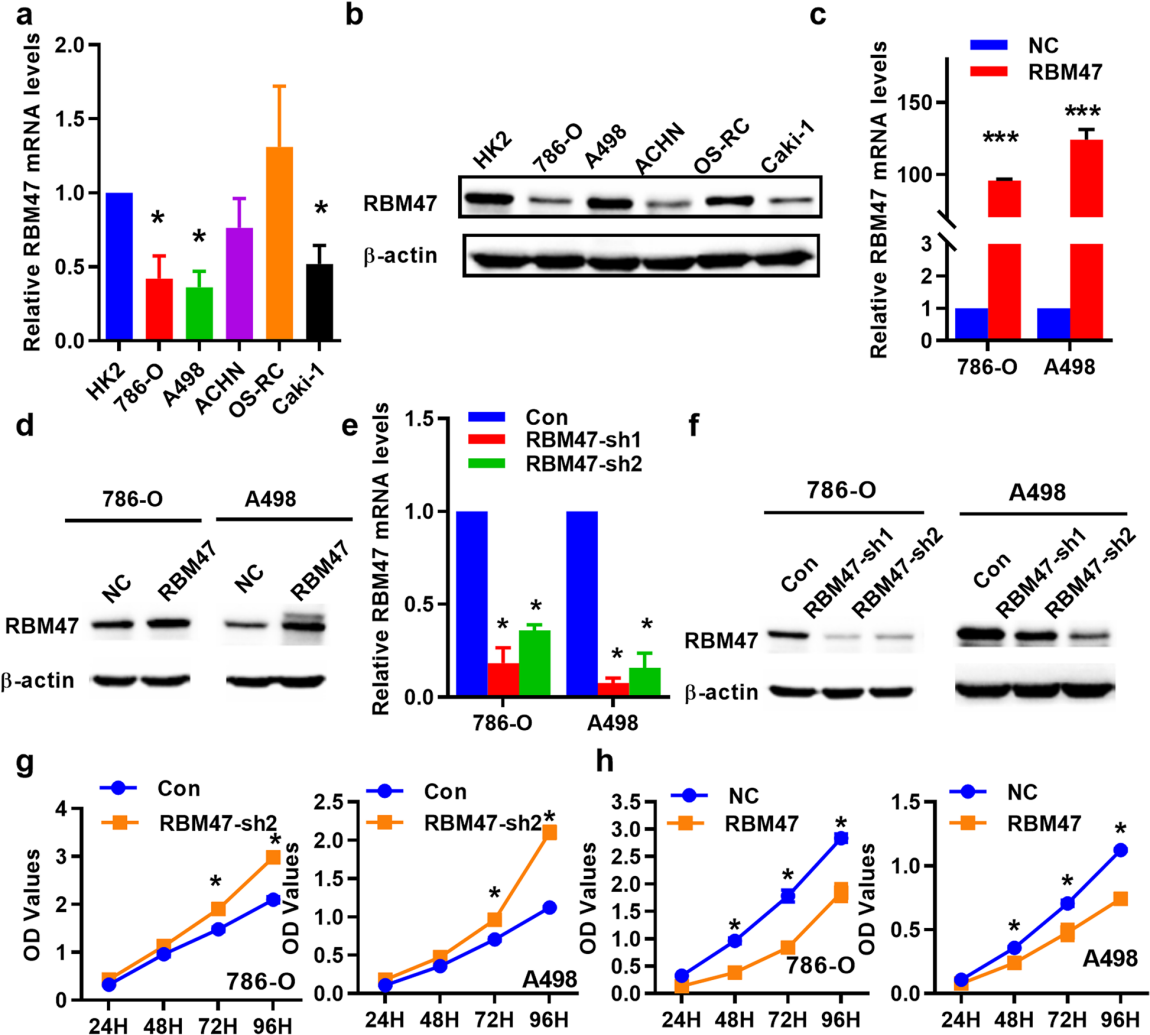
RBM47 inhibited the proliferation of renal cancer cells. **a** and **b** The mRNA and the Protein levels of RBM47 were reduced in renal cancer cells. **c** and **d** Successfully transfected the plasmid of overexpressing RBM47 into renal cancer cell lines. **e** and **f** Successfully knocked down RBM47 in renal cancer cell lines. **g** and **h** RBM47 knockdown promoted while overexpression repressed proliferation of 786-O and A498 cells by cell counting kit-8 assays (*n* = 4). Data were showed with means ± standard. *P < 0.05, **P < 0.01

Subsequently, we verified the expression and role of RBM47 in renal cancer cells, as it can serve as a prognostic marker for renal cancer patients. The results showed the mRNA and the protein levels of RBM47 were down-expressed in renal cancer cells (Fig. 3a and b). Then, we overexpressed or knockdown RBM47 with plasmid in 786-O and A498 cells (Fig. 3c and f). Knockdown RBM47 promoted the proliferation ability of renal cancer cells (Fig. 3g). In contrast, the proliferation ability was inhibited when RBM47 overexpression (Fig. 3h). These results showed that the expression of RBM47 was decreased in renal cancer cell lines when compared with renal tubules, and its reduction could promote the growth of renal cancer cells, while its overexpression had the opposite effect.

### RBM47 affects cell invasion and migration of renal cancer

**Fig. 4:**
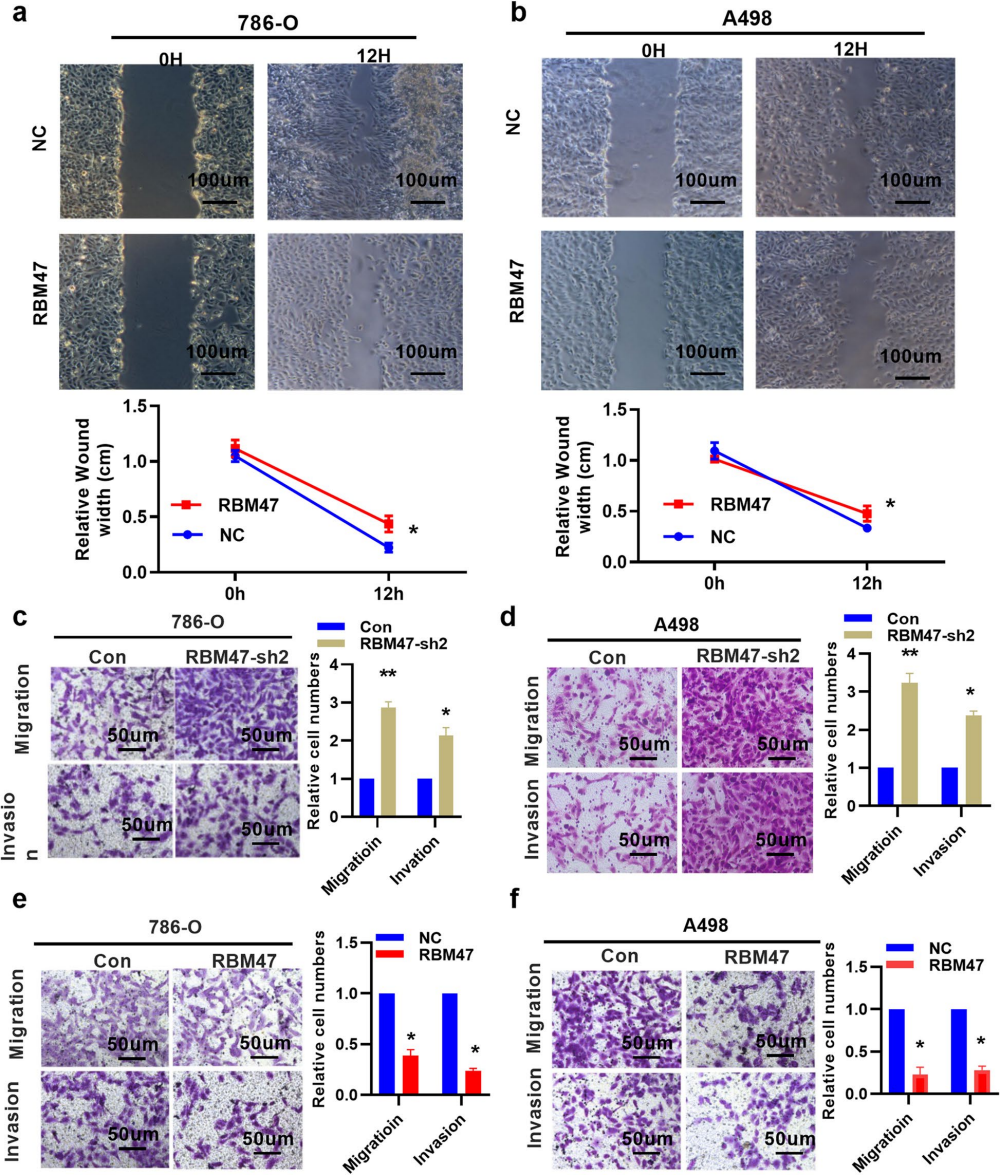
RBM47 overexpression inhibited cancer cell malignant features. **a** and **b** Overexpressing RBM47 inhibited healing ability in renal cancer cells (*n* = 3). **c** and **d** Knockdown RBM47 enhanced cell migration and invasion in 786-O and A498 cell (*n* = 3). **e** and **f** Overexpression of RBM47 repressed cell migration and invasion in 786-O and A498 cell (*n* = 3). Data were showed with means ± standard. *P < 0.05, **P < 0.01

Next, we studied the invasion and migration role of RBM47 on renal cancer cells by plasmid transfection, as it could inhibit the growth of renal cancer. RBM47 overexpression plasmid transfection could inhibit renal cancer cell wound-healing ability (Fig. 4a and b). Knockdown RBM47 promoted cell invasion and migration by transfection of RBM47 shRNA-2 (RBM47 sh-2) in RCC cells (Fig. 4c and d). Transfection with plasmid of overexpressing RBM47 significantly suppressed invasion and migration in RCC cells as shown by transwell assays (Fig. 4e and f). The experimental results indicated that overexpression of RBM47 could inhibit tumor cell invasion and migration ability.

### Signaling pathway of RBM47 calculation in renal cancer

**Fig. 5:**
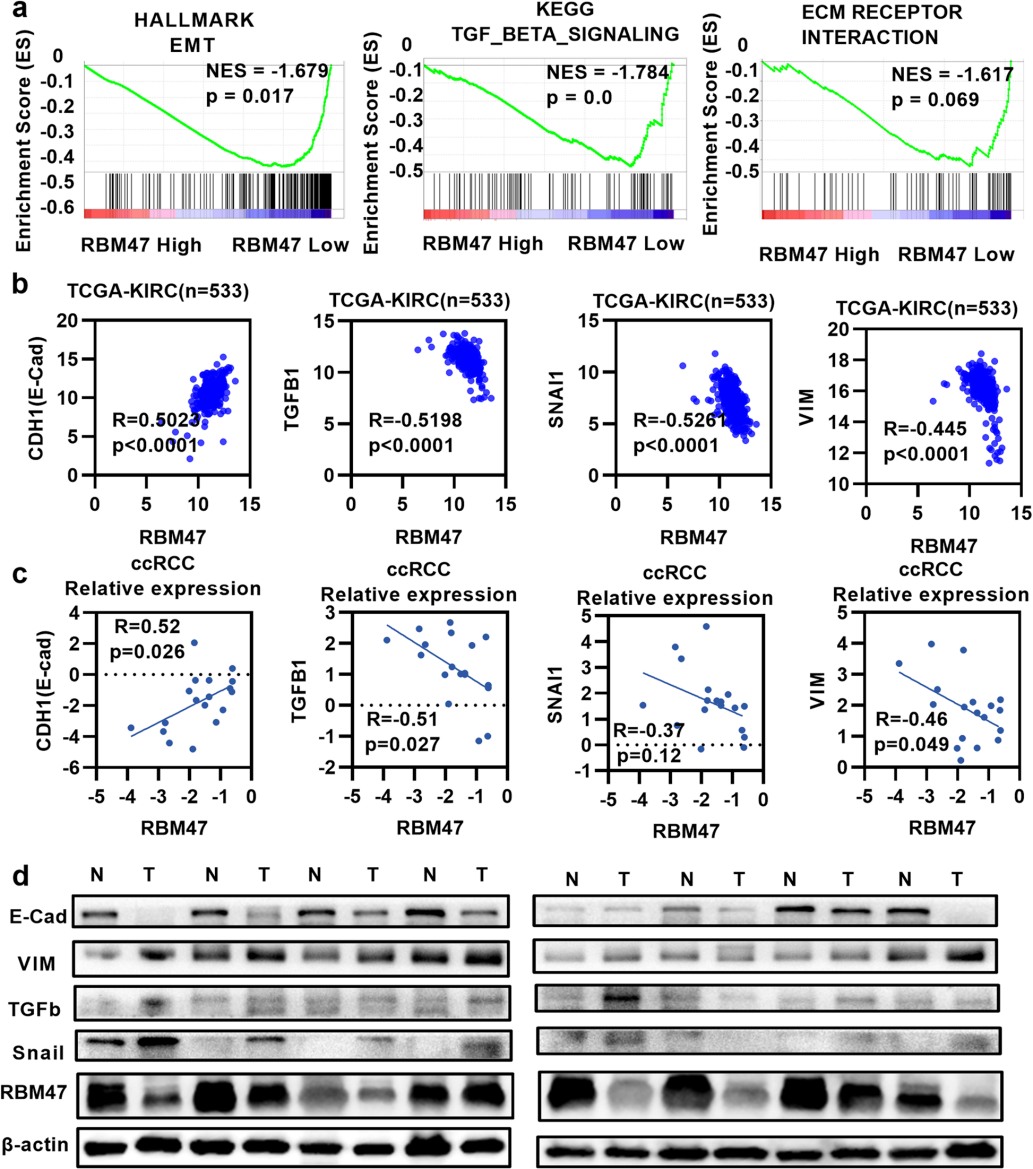
The signaling pathways and correlations involved of RBM47 in ccRCC. **a** GESA showed RBM47 had a negative correlation with EMT, the TGF-β signaling pathway, and ECM receptor interaction. **b** and **c** RBM47 had a negative correlation with TGF-β, snail, and vimentin, and a positive correlation with e-cadherin in TCGA-KIRC data and clinical samples. **d** Protein level relationship of RBM47 and snail, e-cadherin, vimentin, and TGF-β in clinical samples. GESA: Gene set enrichment analysis; VIM: vimentin; E-cad: e-cadherin epithelial-mesenchymal transition (EMT)

Subsequently, we investigated the signaling pathways that RBM47 may be involved in as it can inhibit the malignant features of renal cell carcinoma. Gene set enrichment analysis (GSEA) predicted the biological pathways which RBM47 might be involved in. The results showed that ECM receptor interaction, the transforming growth factor-β signaling pathway, and epithelial-mesenchymal transition (EMT) might be involved in pathological processes of ccRCC (Fig. 5a). Then, we explored the correlation of RBM47 with VIM, TGF-β, e-cadherin, and snail in the TCGA-KIRC (Fig. 5b). The same results were verified in renal cancer tissues, with a positive correlation between RBM47 and CDH1(e-cadherin), a negative correlation between RBM47 and TGF-β, snail, vimentin by qRT-PCR (Fig. 5c), and western blot (WB) (Fig. 5d). These results suggested that RBM47 may inhibit the malignant ability of renal cell carcinoma through the EMT signaling pathway.

### Validation of RBM47 and EMT pathway in renal cancer cells

**Fig. 6:**
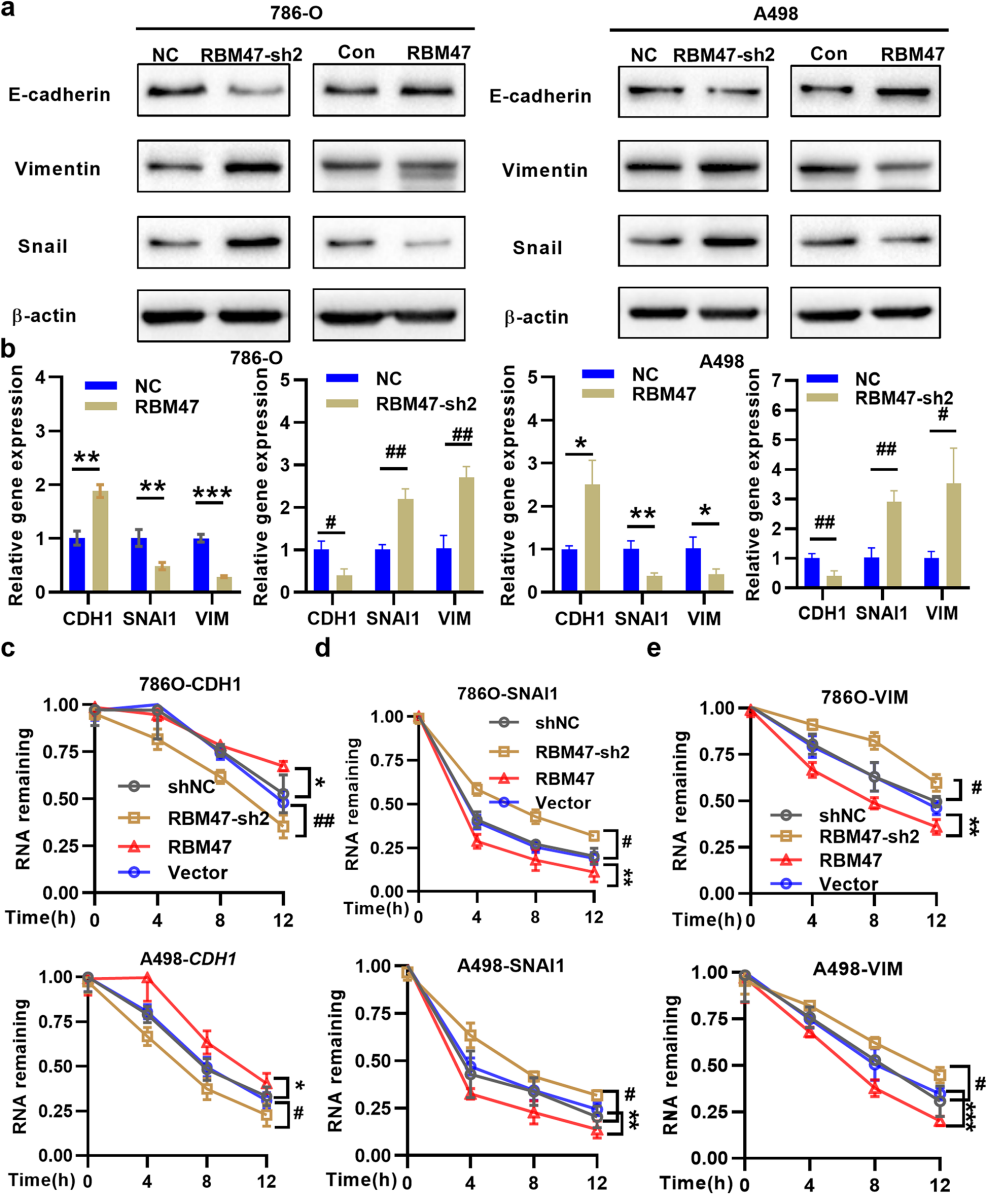
RBM47 interfered renal cancer cells by RNA stability modification via EMT signaling. **a** and **b** Knockdown RBM47 repressed e-cadherin but increased snail and vimentin, overexpression RBM47 repressed snail and vimentin but increased e-cadherin in 786-O and A498 cell (*n* = 3). **c-e** The effects of RBM47 knockdown or overexpression on mRNA remaining of CDH1(e-cadherin), snail, and vimentin after treatment with actinomycin D (2 µg/ml) at the indicated time points (0, 4, 8,12 h) in 786O and A498 cells (*n* = 3). VIM: vimentin, CDH1: e-cadherin, RBM47 overexpression VS vector, *P < 0.05, **P < 0.01, ***P < 0.001, Knockdown VS shNC, *P < 0.05, **P < 0.01

At last, we verified the relationship of RBM47 and EMT pathway in renal cancer cells by qRT-PCR and WB. The results represented that knockdown RBM47 reduced e-cadherin and facilitated snail and vimentin in renal cancer cells while RBM47 overexpression achieved the opposite results (Fig. 6a and b). And, we investigated how RBM47 regulated EMT signaling in renal cancer cell lines. RNA decay experiments indicated that RBM47 silencing decreased e-cadherin mRNA but stabilized the vimentin or snail mRNA in 786O and A498 cells (Fig. 6c and e). RBM47 increased the instability of e-cadherin mRNA, but might not directly regulate vimentin or snail.

## Discussion

CcRCC is a fatal malignant tumor in urology, with high metastasis and mortality rates [8]. Previous studies have shown that hypoxia-inducible factor 2 [30], transient receptor potential (TRP) ion channel 2 [31] could facilitate tumor progression by relieving endoplasmic reticulum stress (ERS) in clear cell renal cell carcinoma. ERS upregulated methyltransferase-like 3 and then downregulated the stability of downstream gene in preeclamptic placentas [32]. This study reported the clinical significance, expression, and biological function roles, ability to regulate RNA stability of RNA-binding protein (RBP) (RBM47) in ccRCC for the first time.

RBP could regulate gene transcriptome through various mechanisms such as transcriptional stability, variable polyadenylation, and selective splicing [11]. Numerous evidence indicated that RBP had an important role in various cancers [33]. HNRNPC, an RNA binding protein, was proved to interact with circRNA to promote its biogenesis [34]. RALY, another RNA binding protein, could activate the cholesterol synthesis in hepatocellular carcinoma [35]. RBM10 could regulate the alternative splicing of transcripts [36]. Overexpression of RBM17 inhibited the sensitivity of human hypopharyngeal cancer cells to cisplatin and promote cell invasion [37]. RBM24 stabled PTEN mRNA and repressed colorectal tumorigenesis in a previous study [38].

RBM47 could inhibit PTC cell proliferation by directly binding and stabilizing SNHG5 [19]. RBM47 might bind to Nanog mRNA with three RRM domains [39]. The downregulation of RBM47 could regulate the occurrence of intestinal tumors by regulating proliferative inflammation and oxidative response pathways [40]. Transfection of exogenous RBM47 were used to analyze and validate the mRNA binding to RBM47 [18]. RBM47, as a transcription factor and selective splicing regulator, promoted the development of nasopharyngeal carcinoma through various pathways [41]. Previous studies have not reported the role of RBM47 in renal cell carcinoma, and this study report the role of RBM47 in ccRCC for the first time.

Bioinformatics analysis and research results prompted that RBM47 was reduced in ccRCC cancer tissues in TCGA-KRIC, ICGC, GEO, CPTAC, and clinical samples, its low expression indicated poor prognosis in ccRCC. Furthermore, RBM47 overexpression inhibited the progression of ccRCC, whereas RBM47 knockdown enhanced the proliferation and malignant features of renal cancer cells. Then, GSEA showed RBM47 might be involved in the biological pathways of EMT and TGF-β. As TGF-β signaling could induce EMT in cholangiocarcinoma [42, 43] and hepatic stellate cells [44], we incorporate the functional of RBM47 on EMT signaling. Previous study had shown the downregulation of RBM47 could promote colorectal cancer progression through EMT [15]. We found that RBM47 overexpression inhibited EMT of renal cancer cells. Mechanically, RBM47 could directly modify RNA stability of e-cadherin, but indirectly regulate snail, vimentin in renal cancer cells. The innovation and value of this article lies in the completeness of the combination of analytical and experimental data.

Although we have obtained the inhibitory effect and mechanism of RBM47 on renal cell carcinoma. However, whether it can serve as a clinical indicator for renal cell carcinoma requires further expansion of clinical samples and multicenter clinical data validation. How RBM47 directly or indirectly affects the genetic stability of EMT, and the action or binding sites of RBM47 on EMT makers has not been studied here. Finally, we have not validated our consequents at the level of animal experiments, which is the content of our other research projects.

In conclusion, previous studies showed that RBM47 had the certain clinical implications in various tumors and participated in tumor formation. We validated its expression with small samples and public database in renal cancer, and predicted its survival role at the RNA level in public database. Subsequently, we verified the role of RBM47 in renal cancer cell lines. RBM47 can directly modify the RNA stability of e-cadherin, indicating that it may affect the EMT characteristics of renal cancer cells and serve as a marker of renal cell carcinoma.

## Materials and methods

### Patient sample tissues

All the tissue samples were obtained from Wuhan Union Hospital (China). We collected 46 pairs of tissue samples from 2014 to 2020 and the operation was approved by the Institutional Review Board of Huazhong University of Science and Technology. All patients had informed the consent in line with the Helsinki Declaration as previous research [45].

### Cell and tissue RNA extraction and qRT-PCR

After collecting tissues and cells, we used TRIzol (Thermo Fisher Scientific, Inc., Waltham, MA, USA) to extract total RNA according to the instructions. Then, we measured the RNA concentration with NanoDrop 2000 spectrophotometer (NanoDrop Technologies, USA). Finally, we performed the gene expression with Stepone plus and the SYBR Green mix (Thermo, USA). Relative gene expression was showed to GAPDH: 2^−ΔCt^ (ΔCt = Ct_gene_–Ct_GAPDH_) as previously described [46]. Qinke (Qinke, China) provided the primers:

RBM47 Forward 5’-TGTCATTCCCACTGTGTCGA-3’.

Reverse 5’-GTAGCCTGCGTATCCTCCAT-3’.

GAPDH Forward 5’-GAGTCAACGGATTTGGTCGT-3’.

Reverse 5’-GACAAGCTTCCCGTTCTCAG-3’.

Snail Forward 5’-TCGGAAGCCTAACTACAGCGA-3’.

Reverse 5’-AGATGAGCATTGGCAGCGAG-3’.

TGFβ Forward 5’-GGCCAGATCCTGTCCAAGC-3’.

Reverse 5’-GTGGGTTTCCACCATTAGCAC-3’.

CDH1 (E-cad) Forward 5’-CGAGAGCTACACGTTCACGG-3’.

Reverse 5’-GGGTGTCGAGGGAAAAATAGG-3’.

Vimentin Forward 5’-GACGCCATCAACACCGAGTT-3’.

Reverse 5’- CTTTGTCGTTGGTTAGCTGGT-3’.

### Actinomycin D treatment and mRNA stability assay

For the mRNA stability assay, blocked gene transcription of 786O and A498 cell with actinomycin D (2 µg/mL) (Merck, Germany). The remaining RNAs were extracted and analyzed after blocking 0, 4, 8, or 12 h, as previously reported [28].

### Immunohistochemistry (IHC)

CcRCC cancer and adjacent normal tissues were collected, fixed in formalin for 24 h, subjected to gradient dehydration, paraffin embedding, antigen repair, and then incubated with the primary antibody of RBM47 (1:100; A14394; ABclonal, China) at 4 °C overnight. Next day, incubated the slice at 37 °C for 1 h, washed 3 times and incubated the secondary antibody (1:200; GB23303; Servicebio, USA) at 37 °C for 2 h as previously described [47].

### Cell culture

The renal cancer cell A498, ACHN, 786-O, CAKI were purchased from from the American Type Culture Collection (ATCC) (Manassas, USA), while OSRC-2 purchased from National Infrastructure of Cell Line Resource (Shanghai, China). Human kidney-2 (HK2) were purchased from the American Type Culture Collection (ATCC) (Manassas, USA). The renal cancer cells and HK2 were cultivated in cell culture medium which contains 10% FBS (FBS; Gibco; Thermo Fisher Scientific, USA) and high-glucose DMEM (HyClone; USA) in a 5% CO_2_ incubator at 37 °C.

### Cell transfection

Next, we knocked down the RBM47 protein by transfecting RBM47 (sh-RBM47) including sh-RBM47-1, sh-RBM47-2, and sh-RBM47-3, while contrasted with negative control (CON). Overexpressing RBM47 protein with RBM47 overexpression plasmid, while contrasted with negative control (NC) from GeneChem (Shanghai, China). Transfected 3 µg plasmid of RBM47 or NC, sh-RBM47 or CON to renal cancer cells in cell experiments by Lipofectamine® 2000 reagent (Thermo Fisher Scientific, USA) [29].

### Cell proliferation analysis

Renal cancer cells were cultured in 6-well plate and transfected with sh-RBM47 or CON, RBM47 or NC with 48 h. Next, cells were collected, resuspended, counted, and seeded in a 96-well plate with 2 × 10^3^ cells for 24, 48, 72, 96 h. We calculated the cell proliferation rate with OD value 1 h later at 450 nm by cell counting kit-8 (CCK-8) (Dojindo Molecular Technologies, USA).

### Wound-healing, migratory and invasion of cancer cell

Renal cancer cells were cultured and transfected with RBM47 or NC with 48 h in 6-well plates, wounded with a 10‐µL pipette tip, imaged the cells at 0, 12, and 24 h. For migration assays, we cultured the cancer cells with serum‐free medium for 24 h after RBM47 or NC transfection. Then, seeded 2 × 10^4^ cells in each top chamber of the insert (Corning Incorporated, USA) with serum-free medium. While covered the top chamber membrane with Matrigel (Thermo Fisher Scientific, USA) overnight at 4 °C, then seed cancer cells with 4 × 10^4^ cells / well at room temperature in invasion assays. At last, erased the upper layer cells on top chamber membrane, fixed 100% methanol, stained 0.05% crystal violet to the deep cell after 24 h. Counting cells numbers with three independent times with microscope (Olympus, Japan) as previous study [29].

### Western blotting

Add liquid ammonia to the collected sample tissue, grind and crush the tissue through a grinding bowl, add it into the EP tube, and put it into − 80 °C for frozen storage. Take a few samples for protein extraction with cell lysate. Quantified the extracted protein with bicinchoninic acid kit (BCA) (Beyotime Institute of Biotechnology, China), diluted to the same concentration, denaturated the proteins, and stored them in -20 °C for frozen storage. 30 µg of protein was separated through gel electrophoresis and transferred to polyvinylidene fluoride (PVDF) membranes (EMD Millipore, Billerica, MA, USA). Blocked PVDF membranes with PBS containing 5% nonfat milk, incubated with primary antibodies (1:1,000) against RBM47, E-cadherin, Vimentin, TGF-β, snail (ABclonal Biotech, China), and next day with secondary antibodies (1:10,000) (BA1020; Boster Biological Technology, China), detected the proteins with ChemiDoc-XRS+ [48].

### Statistical analyses

Paired sample t-test was used to analyze two paired samples, while unpaired samples with unpaired sample t-test. Three or more samples were analyzed with one-way ANOVA test. Kaplan–Meier (KM) curve showed the survival rate of TCGA-KIRC with RBM47 expression level with disease free survival (DFS, 418 samples) and overall survival (OS, 515 samples). 515 samples, with all clinical information (including all TNM stage grade, T, N, M) were used for analysis of the correlation between clinicopathological parameters, prognostic significance with RBM47 by SPSS Statistics 23.0 (IBM Corporation, USA). it is considered statistically significant when P < 0.05 [49].

### Bioinformatics analysis

The Cancer Genome Atlas contains a variety of tumor clinical databases, and the data including 533 cases of Clear cell renal carcinoma (TCGA-KIRC) can be downloaded through the Xena Functional Genomics Explorer (XFGE) [50]. We analyzed the signaling pathways that RBM47 may influence in renal cell carcinoma through Gene set enrichment analysis (GSEA) [51]. P < 0.05 and false discovery rate (FDR) of < 25% and were considered to be significantly [52]. The protein levels of ccRCC samples were obtained from the Clinical Proteomic Tumor Analysis Consortium (CPTAC) [51]. Gene Expression Omnibus (GEO) databases [53] and International Cancer Genome Consortium (ICGC) [54, 55] were used to verify the expression of RBM47 in clinical samples.

### Supplementary Information

**Additional file 1.**

## Data Availability

All data presented were contained within the manuscript, and were available from the corresponding author on reasonable request.
