# Supplementary material for: Downregulation of RNA binding protein 47 predicts low survival in patients and promotes the development of renal cell malignancies through RNA stability modification
Source: Mol Biomed. 2023 Nov 14;4:41. doi: 10.1186/s43556-023-00148-w (PMC10645769; doi:10.1186/s43556-023-00148-w)
Supplement: Supplementary file 1 — Additional file 1. [file 43556_2023_148_MOESM1_ESM.docx]

Downregulation of RNA binding protein 47 predicts low survival in patients and promotes the development of renal cell malignancies through RNA stability modification

Cheng Wang^1,2,#^, Weiquan Li^1,2,#^, Xiangui Meng^1,2,#^, Hongwei Yuan^1,2^, Tiexi Yu^1,2^, Wei Yang^1,2^, Dong Ni^1,2,^*, Lei Liu^1,2,^*, Wen Xiao^1,2,^*

^1^ Department of Urology, Union Hospital, Tongji Medical College, Huazhong University of Science and Technology, Wuhan 430022, China

^2^ Institute of Urology, Union Hospital, Tongji Medical College, Huazhong University of Science and Technology, Wuhan 430022, China

# These authors contribute equally to this study.

*Correspondence author:

Wen Xiao, Department of Urology, Union Hospital, Tongji Medical College, Huazhong University of Science and Technology, Wuhan, 430022, China; E-mail: [wxuro20@hust.edu.cn](mailto:xzhang@hust.edu.cn)

Lei Liu, Department of Urology, Union Hospital, Tongji Medical College, Huazhong University of Science and Technology, Wuhan, 430022, China; E-mail: liulei2016@hust.edu.cn

Dong Ni, Department of Urology, Union Hospital, Tongji Medical College, Huazhong University of Science and Technology, Wuhan, 430022, China; E-mail: dni@hust.edu.cn


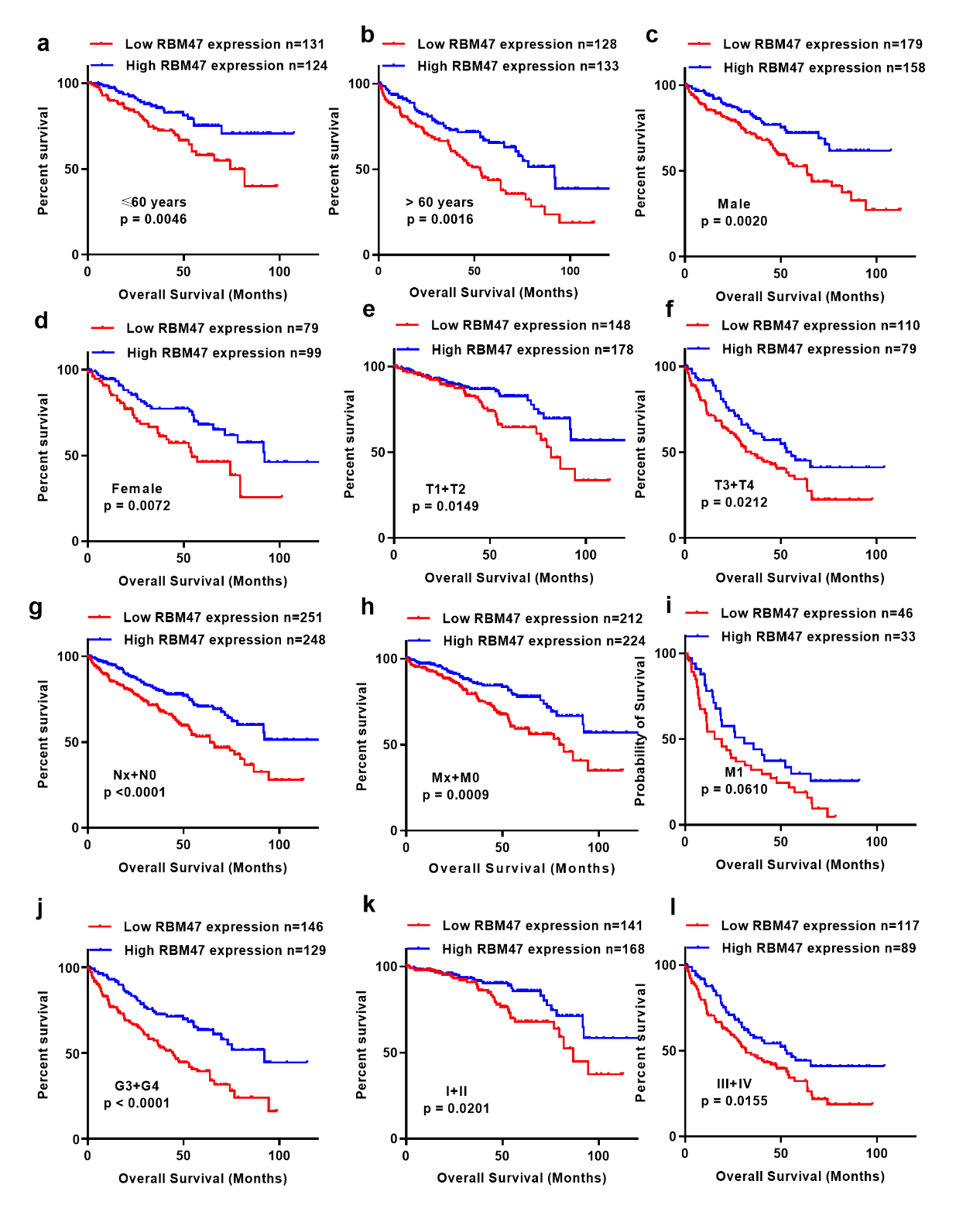


Supplementary Figure 1. Low expression of RBM47 predicts poor overall survival in subgroups of ccRCC patients. Subgroups of ccRCC patients were compared with RBM47 mRNA to overall survival analysis: (a) Age ≤ 60 years, (b) Age > 60 years, (c) male, (d) female, (e) T1+T2, (f) T3+T4, (g) N0 stage, (h) non-metastasis status, (i) metastasis status, (j) G3+G4 stage, (k) TNM I+II stage, (l) TNM III+IV stage.


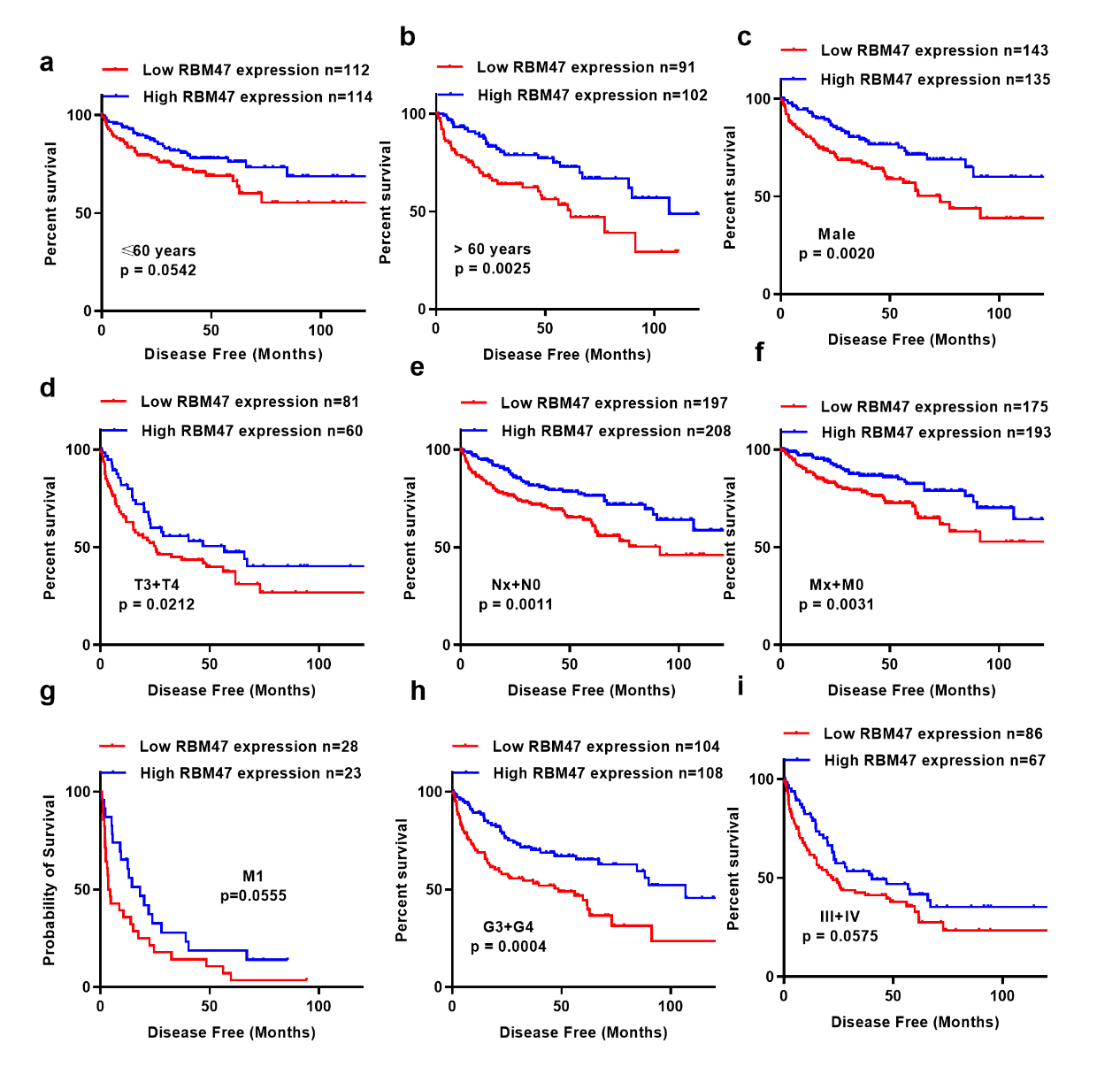


Supplementary Figure 2. Low expression of RBM47 predicts poor disease-free survival in subgroups of ccRCC. Kaplan-Meier was used to analyze the correlation between RBM47 expression and disease-free survival time: (a) Age ≤ 60 years, (b) Age > 60 years, (c) male, (d) T3+T4, (e) N0 stage, (f) non-metastasis status, (g) metastasis status, (h) G3+G4 stage, (i) TNM III+IV stage.
